# Resistive switching in diamondoid thin films

**DOI:** 10.1038/s41598-020-76093-3

**Published:** 2020-11-04

**Authors:** A. Jantayod, D. Doonyapisut, T. Eknapakul, M. F. Smith, W. Meevasana

**Affiliations:** 1 Department of Physics, Faculty of Science, Naresuan University, Phitsanulok, 65000 Thailand; 2 School of Physics, Suranaree University of Technology, Nakhon Ratchasima, 30000 Thailand; 3 Center of Excellence on Advanced Functional Materials, Suranaree University of Technology, Nakhon Ratchasima, 30000 Thailand; 4 Thailand Center of Excellence in Physics (ThEP), MHESI, Bangkok, 10400 Thailand

**Keywords:** Materials science, Physics

## Abstract

The electrical transport properties of a thin film of the diamondoid adamantane, deposited on an Au/W substrate, were investigated experimentally. The current *I*, in applied potential *V*, from the admantane-thiol/metal heterstructure to a wire lead on its surface exhibited non-symmetric (diode-like) characteristics and a signature of resistive switching (RS), an effect that is valuable to non-volatile memory applications. *I*(*V*) follows a hysteresis curve that passes twice through $I(0)=0$ linearly, indicating RS between two states with significantly different conductances, possibly due to an exotic mechanism.

## Introduction

Resistive switching (RS) is a phenomenon where the electrical resistance *R* of a material or junction changes reversibly, typically between two values, in response to a strong applied electric field^1,2^. The current–voltage (*I*–*V*) curve follows a hysteresis loop with a switch between the high-resistance state (HRS) and low-resistance state (LRS) occurring at voltage extremes and two different values of *I* measured at the same *V* on alternate passes around the loop. There has been longtime interest^3,4^ in the physical mechanism of RS and research has also been driven by the potential for applications to non-volatile memory (NVM) in computers^5,6^.

NVM retains information even when no power is provided to the device. NVM based on magnets, ferroelectrics, phase-change materials, photonics and many other mechanisms have been realized^7–14^. It is natural to employ the resistive switching effect in NVM devices: the LRS and HRS can serve as the “on” and “off” states for a memory bit.

Resistive-switching has been seen in many materials and heterostructures^15^, mainly involving metal-oxides^16–18,21–24^ but also nitrides^25,26^, chalcogenides^27,28^ and other inorganic families^29^. It has been seen in organic-based junctions as well^30–32^.

For the insulating oxide, RS occurs because the applied electric field redistributes defects (mainly oxygen vacancies) to form conducting filaments that run through the sample^4,33–35^. In the LRS, the resistance is determined by these filaments and the system behaves like a metal. A strong field, and large current, causes the filaments to rupture and the system returns to its natural insulating state, the HRS. This cycle repeats during an observation of the hysteresis loop. There are other mechanisms^3,19–21^, appropriate for RS in tunneling currents through extremely thin films, that are somewhat analogous to this picture. The field can displace defects that enable trap-assisted tunneling conduction, for example.

Recently, there have been attempts to apply RS effects in two-dimensional materials, notably those based on graphene, to NVM^23,36^. The advantages of a 2D material in terms of storage density are obvious, and carbon-based materials, generally, tend to have material advantages like high chemical stability and low cost.

Diamondoids are a family of hydrocarbon materials in which the carbon atoms belonging to cyclohexane rings are arranged in the diamond crystal lattice^37^. They can be extracted from the waste products of petroleum processing. When diamondoid is introduced to the surface of Au, a monolayer self-assembles on the metal surface^38,39^. That is, the resulting film is one molecule (roughly 1nm) thick. These monolayers have a number of promising electronic properties^40,41^, such as a negative electron affinity^42^ (a property they share with the surface of diamond^43^) and an ultra-low work function^44^. We are not aware of any work done on RS in diamondoid monolayers with a view to NVM applications.

We were thus motivated to study the transport properties of diamondoid thin films on an Au surface, particularly adamantane, which is the smallest 1-cage member of the diamondoid series. We found that RS occurs for the current measured between a diamondoid film and an Au/W substrate. The *I*–*V* curve follows a robust hysteresis loop, with the LRS and HRS seen at low voltage on alternate passes. When the junction is in the LRS and we disconnect it from the current source and wait for some time, it retains its previous resistance after being reconnected.

This paper reports the RS we see in the current through a thin diamondoid film on a metallic substrate. The LRS and HRS for the film are typically Ohmic at room temperature, with resistances differing by seventy percent. While the similarity of the LRS and HRS is not ideal for NVM, it appears that the mechanism for RS could be unusual. We present our finding because it may interest those studying the fundamental physics of RS, and could be a first step towards NVM devices based on the observed effect.

## Experimental

The *I*–*V* curves were studied using the cat whisker setup. The current through a cat whisker, which was the name of an early design of the semiconductor diode, passes from a semiconducting material to a thin wire contacting its surface. For a surface of diamondoid (adamantanethiol) films on Au/W substrate, the current *I* versus voltage *V* showed non-symmetric (diode-like) behavior and the signature of resistive switching.

There is a well-established procedure for fabricating diamondoid monolayers via self-assembly on the surface of gold and other noble metals (e.g. Ref.^38^). Following this procedure, diamondoid films were prepared starting from a precursor of adamantane powder with a purity of 99.0$\%$, via the self-assembled monolayer (SAMs) technique. A $5\times 5$ mm$^2$ tungsten plate in ethanal was cleaned with an ultrasonic bath for 5 min at room temperature. Gold was then coated on the plate using plasma sputtering in an argon atmosphere at 8 Pa for 5 min. All SAMs were fabricated on Au/W substrates immersed in 10mM 1-adamantanethiol solution for 24 h. 10 mM 1-adamantanethiol solution was prepared by inserting 1-adamantanethiol 0.1683 g in 9:1 toluene/ethanol (volume:volume) with final volume of 100 ml. After 24 h, we took a sample out from the solution and washed it with toluene to remove the unbounded molecules. We rinsed it with ethanol and dried it with warm air. A crude, preliminary characterization of the adamantanethiol films was done by rinsing deionized water on the sample to confirm surface hydrophobic properties. Images of the sample surfaces, from atomic force microscopy (AFM) and scanning electron microscopy (STM), are included in the appendix below.

**Figure 1:**
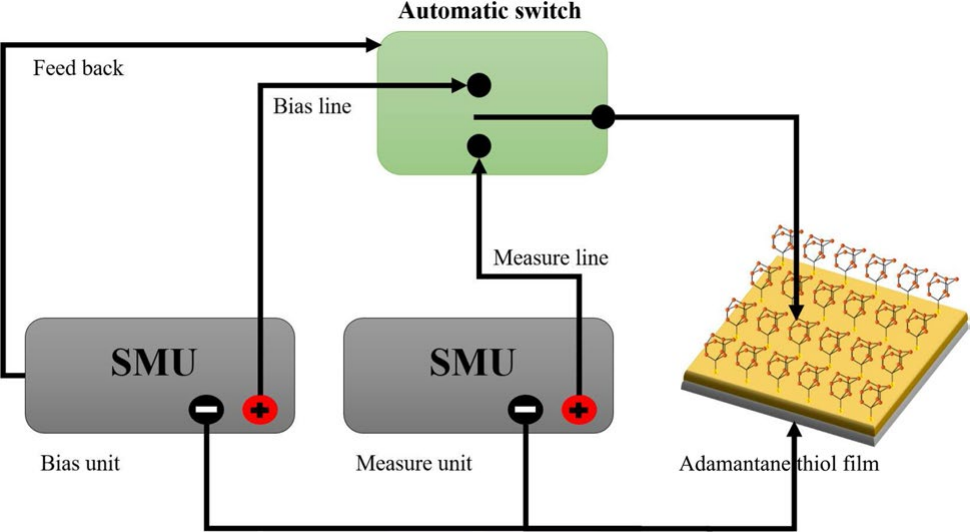
Schematic of the experiment used to measure *I*–*V* curves. A current *I* is driven through the sample, a diamondoid (adamantanethiol) film on an Au/W substrate, when the bias voltage $V=V(t)$ is applied. The bias was ramped up and down in a sawtooth pattern, sweeping from $-1.0$ to 1.0 V and back in 9.2 s. To test the memory effect of the resistive switch, the applied bias was abruptly switched off, and *V* maintained at zero for time $t_s$, before it was switched back to its previous value and the sawtooth variation *V*(*t*) resumed.

The cat-whisker setup (see Fig. 1) employed a source measure unit (SMU) connected to a 200 $\upmu$m diameter copper wire mounted on an adjustable microstage. The wire was moved towards the sample surface, by 10 $\upmu$m increments, until a stable current from the sample through the wire was observed under an applied bias potential *V*. (The voltage *V* is that between the sample surface and cat whisker, which dominates the effective resistance of the circuit). With electrical contact established, we swept *V* from $-1.0$ to 1.0 V and back repeatedly. The voltage increments $\Delta V=0.01$ V were made with each time step $\Delta t=0.02$ s, with a complete cycle taking 9.2 s (including the 3 ms used to ramp up by $\Delta V$ each step). We measured the current *I* throughout this cycle to trace the *I*(*V*) curve.

## Results and discussion

**Figure 2:**
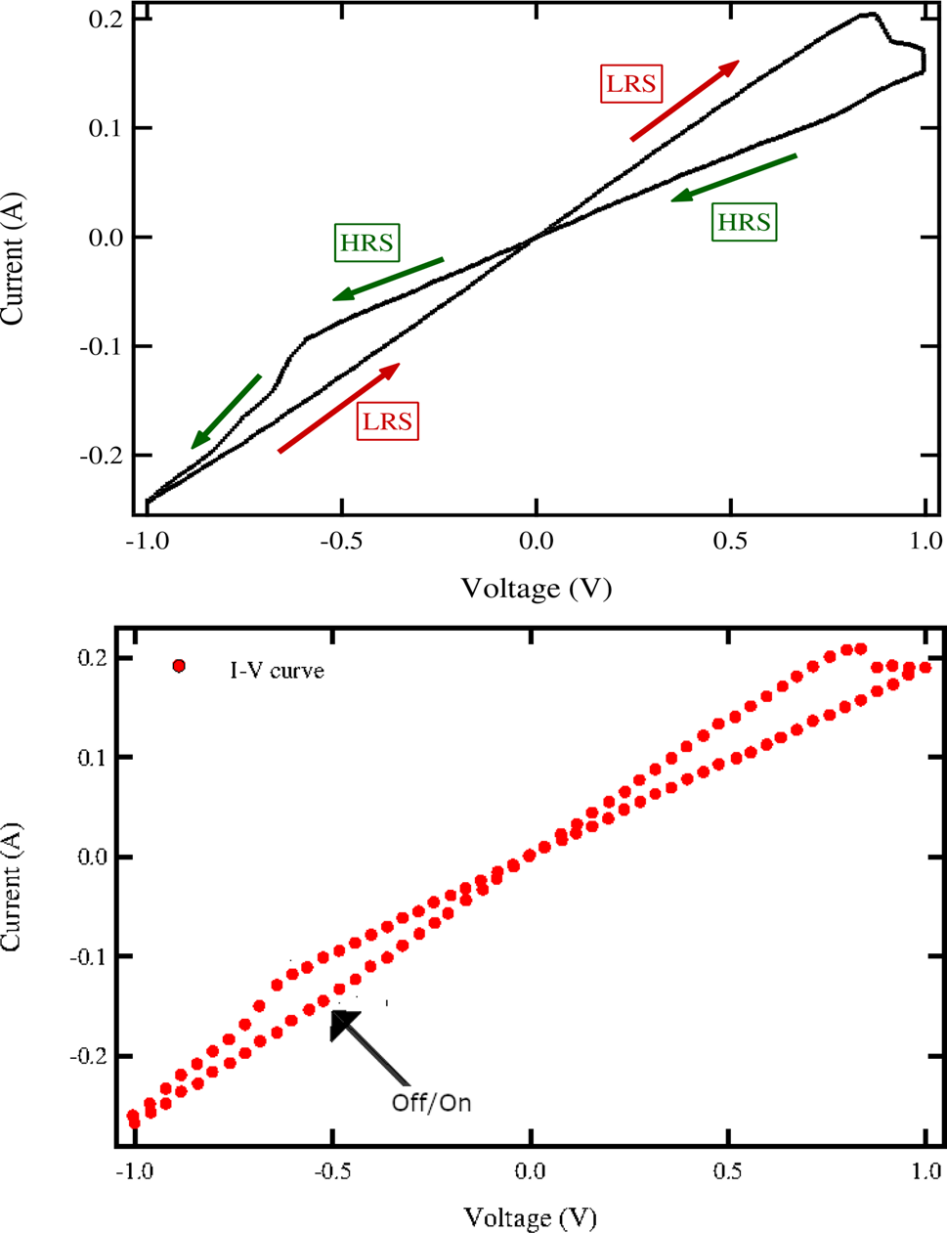
The current–voltage curve *I*(*V*) measured for the adamantanethiol film on an Au/W substrate. (Top) The voltage *V*(*t*) was ramped up and down five times, with the direction of time indicated by arrows, and the average current shown. *I*(*V*) passes twice per cycle through $V=I(0)=0$ with two different conductances. The low-resistance state (LRS) and high-resistance states HRS are thus identified. (Bottom) To test the longevity of the LRS, we interrupted the cycling of *V*(*t*) at the point of the arrow, when $V(t)=-0.5$ V in the LRS: switching off the power and waiting for 5 s before switching it back on and continuing its previous time-variation. The resulting *I*(*V*) curve continued along the previous path, little-affected by interruption, as evident by the qualitative similarity of curves in the top and bottom panels.

Figure 2 (top) shows resistive-switching in the *I*(*V*) curve for the self-assembled diamondoid film on the Au/W substrate. At low *V*, the current *I*(*V*) is linear in *V* but can have two different conductances depending on its recent history. When *V* is increasing, the low-resistance state (LRS) is observed with $I=G_2V$ and $G_2\approx 0.25\,\,\Omega ^{-1}$. When *V* is decreasing, the high-resistance state (HRS) with $I=G_1V$ and $G_1\approx 0.15\,\,\Omega ^{-1}<G_2$ is seen.

Similar hysteresis curves were obtained in measurements on several samples. Note that the *I*–*V* curves shown do not include transient effects seen at the beginning of the measurement, which vary somewhat from sample to sample, but only the reproducible hysteresis loop that establishes itself after ramping the voltage up and down. (The first complete cycle was omitted for these plots, so the first measurement made was at $V=-0.8$ V and they continued for five cycles, with the result shown being the average of these five.) The effect can definitively be attributed to the diamondoid film because we repeated the experiment with a Au/W substrate without the adamantane. The result (see Fig. 5) was a current $I=G_sV$ linear in *V* over the entire range of Fig. 2 with a $G_s\approx 0.4\,\,\Omega ^{-1}$.

The data of Fig. 2 is a typical observation, and the conductance values are representative. The transition from HRS to LRS begins when $V=V_- \sim -0.6$ V and the transition from LRS to HRS when $V=V_+\sim 0.8$ V. The hysteresis curve is robust in the sense that each individual loop lies close to the average shown. Also, RS requires both voltage polarities. The transition between resistive states occurs alternatively at high positive and negative *V*. If one polarity is used, for example if *V* is cycled between 0 and 0.8V while remaining positive, then no RS is seen. These features are illustrated in Fig. 6.

**Figure 3:**
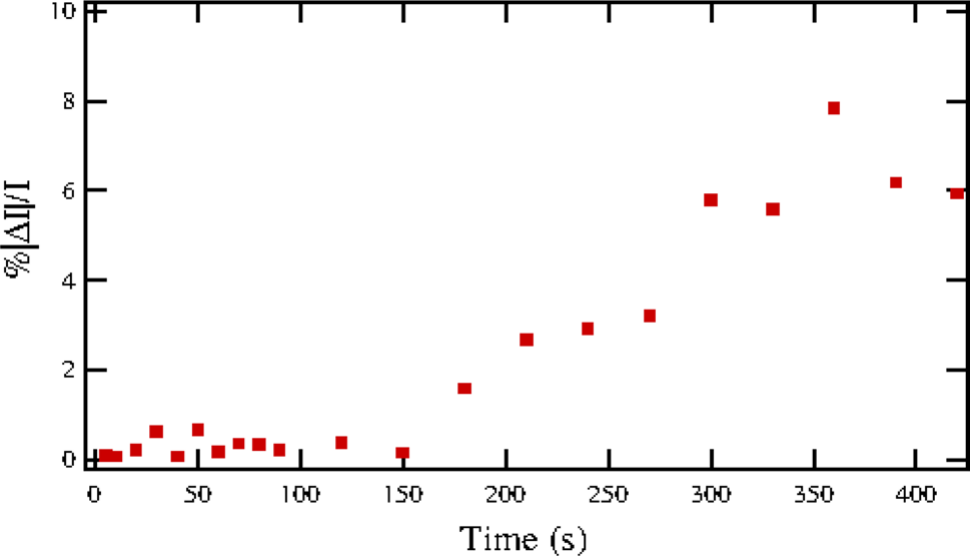
We interrupted the sawtooth variation of the potential *V*(*t*) across the adamantanethiol film on an Au/W substrate. At $t=t_i$ when $V(t_i)=-0.5$ V and rising (the LRS), we switched the voltage off for a time $t_s$, so $V(t)=0$ for $t_i<t<t_i+t_s$, before switching it back on: abruptly restoring *V* to its previous value and resuming its time-variation. The measured current just before $I(t_i)$ and after $I(t_i+t_s)$ the interruption differed by $\Delta I=I(t_s+t_i)-I(t_i)$. The plot, of $|\Delta I|/I(t_i)$ versus $t_s$, suggests that the practical retention time of NVM memory for this configuration is of the order of several minutes.

According to the conventional picture of NVM devices based on RS in oxides, the survival of the LRS, which depends on intact conducting filaments, determines memory retention. We estimated the time scale over which the LRS remains intact by interrupting a sweep of *V*(*t*) at time $t_i$ when the system was in the LRS: disconnecting the potential then waiting for time $t_s$ before resuming the cycle at the point of interruption. In the results show in the bottom panel of Fig. 2 and in Fig. 3, we cycled *V*(*t*) until *V*(*t*) was $V(t_i)=-0.5$ V and rising then abruptly dropped it to zero. After a delay of $t_s$ seconds we resumed the cycle at $V(t_i)$. For the data shown in the lower panel of Fig. 2 we used $t_s=5$s and repeated the sequence five times to obtain the *I*–*V* curve shown. We also measured the difference $|\Delta I|=|I(t_i+t_s)-I(t_i)|$ before and after the interruption, and plotted it versus $t_s$ in Fig. 3.

As seen in the Fig. 2, the *I*–*V* curve resumed its previous course along the hysteresis loop after the voltage was turned back on. So conducting states remain distinct after the 5 s interruption. According to Fig. 3, there is no apparent change in the current (and thus no change in the LRS conductance) for $t_s<150$s but $\Delta I$ increases with larger $t_s$, approaching 10% for $t_s\approx 400$s. In the Appendix, Fig. 9, we show that this same time scale, of the order of a few minutes, is that over which the qualitative hysteresis loop starts to break down.

If the diamondoid films are indeed monolayers, then the conventional picture of extended conducting filaments is not applicable, and a tunneling picture is more appropriate. Since the field perpendicular to the film displaces charged defects, it can change trap energy levels relative to carrier bands and strongly affect the tunneling amplitude. We should assume parallel channels: direct tunneling from the substrate to the electrode alongside indirect tunneling via the diamondoid layer. If the latter is turned on and off by the applied field, this would explain the RS behavior and imply that the system could be modified to greatly enhance the $G_2/G_1$ ratio. Below we follow this line of reasoning in writing down a model that can provide a semi-quantitative description of the observed hysteresis loop.

### Phenomenological model

To understand the *I*(*V*) curve, we start by assuming that there are two states, 1 and 2, associated with each molecular site in the diamondoid film. With all sites in state 1 (or state 2) the total conductance through the film would be ${\tilde{G}}_1(V)$ (or ${\tilde{G}}_2(V)$). An electric field can induce changes between the states. So, under an applied potential *V* at time *t*, a fraction *n*(*V*, *t*) of the sites are in state 1 while the remaining fraction $1-n(V,t)$ are in state 2. This picture is reminiscent of a system with two molecular dipole states, where a strong electric field displaces the charge distribution within the molecule.

The flakes of the diamondoid film are unevenly distributed on the substrate (see Fig. 8), covering an average fraction $\chi$ of its surface. If the cat whisker probe is larger than the typical flake, it will receive a weighted average of two current contributions: one coming through the diamondoid film and the other directly from the bare substrate. The latter can be associated with a conductance $G_s=0.4\,\,\Omega ^{-1}$. The total current is written as

1 $$\begin{aligned} I(V)=(1-\chi )G_sV+\chi G_dV, \end{aligned}$$

where the conductance of the diamondoid film may depend on time and *V* according to

2 $$\begin{aligned} G_d(V,t)=n(V,t){\tilde{G}}_1(V)+[1-n(V,t)]{\tilde{G}}_2(V). \end{aligned}$$

According to the data, the current satisfies Ohm’s law at small *V*, with two different possible values of the conductance: $I=G_1V$ in the HRS and $I=G_2V$ in the LRS. Eq. (1) will give this behavior if one of the dipole states has an extremely small conductance and the other is constant over the voltage range of interest. So, we take ${\tilde{G}}_1(V)\approx 0$ and ${\tilde{G}}_2$ constant. The simplest situation is one in which the fraction *n*(*V*, *t*) undergoes a complete transfer from 0 to 1 over the course of a full voltage sweep. In the LRS $n(V,t)=0$ and $I(V)=([1-\chi ]G_s+\chi G_2)V\equiv G_2V$, while in the HRS $n=1$ and $I(V)=([1-\chi ]G_s)V\equiv G_1V$. With an assumed fractional coverage of $\chi \approx 0.625$, we obtain the measured value $G_1=0.15\,\,\Omega ^{-1}$. The conductance of the active dipole state is set to $\tilde{G_2}=0.16\,\,\Omega ^{-1}$ so that Eq. (1) gives the observed value of $G_2$ as well.

Both the shape of the hysteresis curve and its time dependence are determined by *n*(*V*, *t*). Note that one could modify the interpretation of the model by restricting current flow to a subset of molecular sites, perhaps defect locations, but this would not affect results below. Also, it is clear that this model always predicts zero current when $V=0$, so it cannot account for hysteresis loops that do not pass through the origin, reported elsewhere^45^.

We follow Kramers theory^47^, assuming an effective potential energy *U*(*X*) that depends on a reaction coordinate *X*, related to the dipole moment. Consider independent harmonic potentials for state 1,

3 $$\begin{aligned} U_1(X)=\frac{1}{2}\omega _1^2X^2-qE_0X \end{aligned}$$

and state 2,

4 $$\begin{aligned} U_2(X)=\Delta U+ \frac{1}{2}\omega _2^2(X-\ell )^2-qE_0X \end{aligned}$$

where $E_0$ is a uniform electric field and *q* a charge. Assuming the effective dipole length $\ell$ is of the same order as the thickness of the interface, the potential difference between opposite sides of the film is $V=-E_0\ell$. The level shift $\Delta U$ is constant, $\omega _1$ and $\omega _2$ are harmonic frequencies (with unit mass). We define their ratio as $\eta =\omega _2/\omega _1$, which is presumably of order unity. The basic energy scale $\epsilon _1=\omega _1^2\ell ^2/2$, the energy of a vibration with amplitude equal to the molecular length $\ell$, is expected to be of order 1eV. If we measure $\epsilon _1$ in eV, take $\ell =1$nm, and write for the effective molecular mass $m=2Zm_p$ where $m_p$ is a proton mass, then we have $\omega _1\approx \sqrt{\epsilon _1/Z}10^{13}\mathrm {s}^{-1}$, a typical phonon frequency.

**Figure 4:**
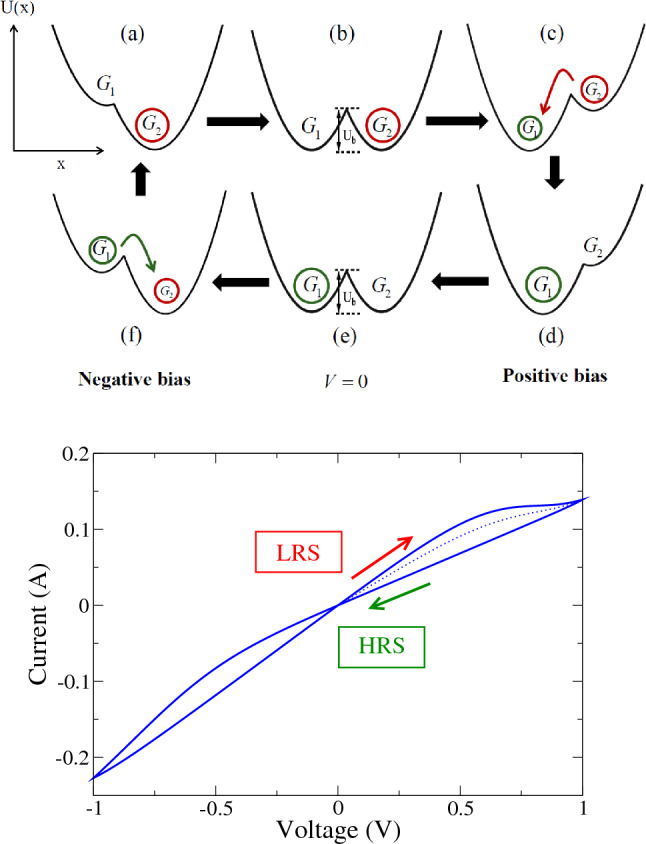
(Top) The model energy *U*(*X*), Eq. (5), for each site on the diamondoid interface is plotted versus the reaction coordinate *X*. The valleys correspond to two stable states: state 1 is responsible for the conductance $G_1$ and state 2 for $G_2$. The potential *U*(*X*) is replotted, following the sequence (a through f), as the voltage *V* is ramped up and down with time. Hysteresis results from changes in the fraction of sites that occupy the two states: there is a population transfer from state 2 to 1 at positive bias $V>0$ and from state 1 to 2 at negative bias $V<0$. (Bottom) The hysteresis curve that results when the voltage is cycled up and down. The dotted segment of the curve shows the initial values of the current, after one half cycle is complete the current loops around the solid curve. Model parameters are discussed in the text.

**Figure 5:**
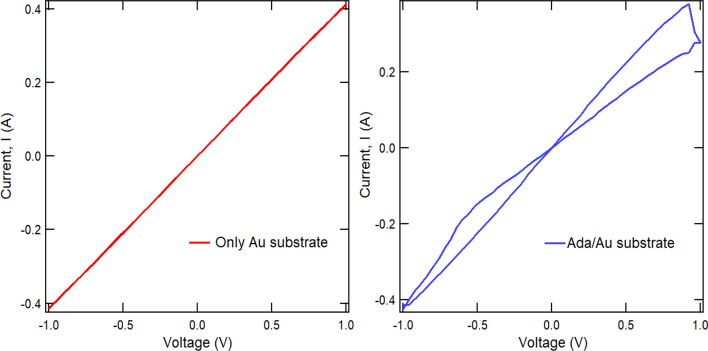
The current *I* versus voltage *V* between the Au/W substrate and the Cu wire lead (the cat’s whisker). On the left panel, the measurement was made using the bare substrate. The current satisfies $I=G_0V$ with $G_s\approx 0.4\,\,\Omega ^{-1}$. On the right, the substrate is coated with a diamondoid layer. The current exhibits the hysteresis loop discussed above.

**Figure 6:**
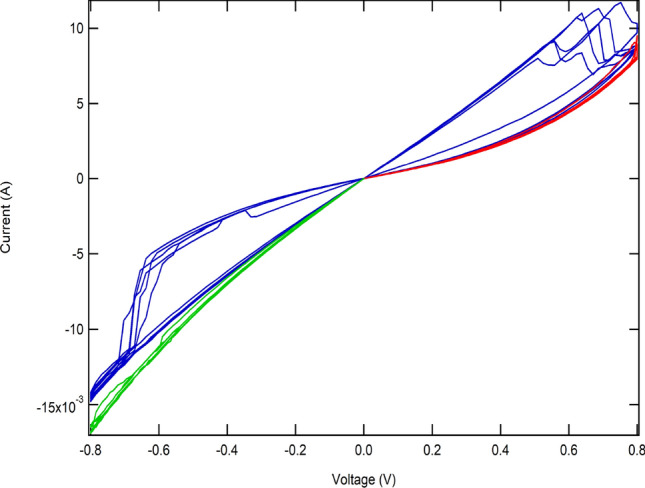
Current *I* versus voltage *V* with the diamondoid film present on the Au/W surface. The blue curve (for non-color plots, the descriptions of curve shape given here should be sufficient) shows *I*(*V*) with *V* starting at zero, ramped up to 0.8 V, down to − 0.8 V with that cycle repeated five times. After the first half cycle it settles into a hysteresis curve similar to those shown earlier. For the red curve, *V* always remained positive: ramped from 0 to 0.8 V and back to zero several times. It shows minimal hysteresis and remains close to the HRS slope. For the green curve, *V* remained negative: 0 to − 0.8 V and back to zero repeatedly. It show minimal hysteresis and remains close to the LRS slope.

**Figure 8:**
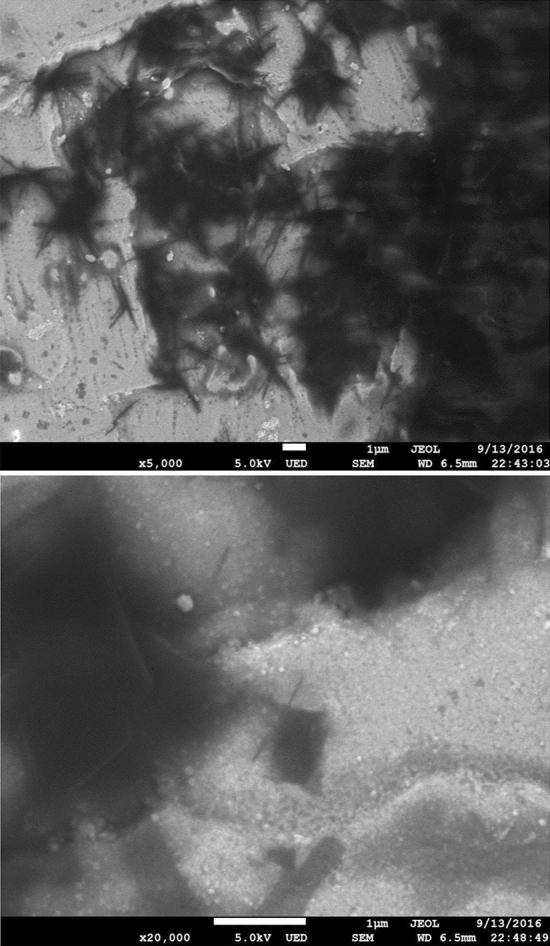
Scanning electron microscopic (SEM) images showing a top-view of the Au/W substrate with the deposited diamondoid thin layer. The diamondoid film appears dark in these images. The visible white bar, corresponding to a length of 1 $\upmu$m indicates the magnification scale.

Weakly coupling $U_1(X)$ and $U_2(X)$ gives a mixed ground state with potential

5 $$\begin{aligned} U(X)=\frac{U_1(X)+U_2(X)}{2}- \frac{|U_1(X)-U_2(X)|}{2} \end{aligned}$$

shown at the top of Fig. 4. The minima are located at $X=X_1=-qV/(2\epsilon _1)$ where the energy is $U(X_1)=U_1(X_1)$, and at $X=X_2=\ell -qV/(2\eta ^2\epsilon _1)$ where the energy is $U(X_2)=U_2(X_2)$. A local maximum, in the form of a cusp, presents an energy barrier at $X=X_b$ between the two valleys. The barrier location is found from $U_1(X_b)=U_2(X_b)$. The relative energies of the two valleys, and the barrier between them, strongly affect the transition rates between states.

The Kramers transition rate from state 1 to 2 is

6 $$\begin{aligned} \Lambda _{1\rightarrow 2}=\omega _1 \exp (-\beta [U(X_b)-U(X_1)]) \end{aligned}$$

where $\beta =1/(kT)$ at temperature *T*. The reverse rate $\Lambda _{2\rightarrow 1}$ is given by the same expressions with $2\leftrightarrow 1$. The fraction $n(t)=n_{eq}+\delta n(t)$ of sites in state 1 changes in time according to:

7 $$\begin{aligned} \frac{d}{dt}\delta n=-\Lambda \delta n \end{aligned}$$

where $\Lambda =\Lambda _{1\rightarrow 2}+\Lambda _{2\rightarrow 1}$. Thus *n*(*t*) relaxes towards its equilibrium value $n_{eq}=n_{eq}(V,T)=\Lambda _{2\rightarrow 1}/\Lambda$.

To simulate the conditions of the experiment, we change the voltage stepwise with time. (We take these step-changes to be instantaneous, so we can still use Eq. (7) between steps.) If the voltage is changed to a value $V_j$ at time $t=t_j$, then the population $n=n(V,t)$ is given, according to Eq. (7), by

8 $$\begin{aligned} n(t)=n_{\mathrm {eq}}(V_j,T)+\delta n(t_j)\exp (-\Lambda [t-t_j])). \end{aligned}$$

where $\delta n(t_j)=n(t_j)-n_{\mathrm {eq}}(V_j,T)$. This dependence continues until the next time, say $t_{j+1}$, that the voltage is changed to $V_{j+1}$, after which we have

9 $$\begin{aligned} n(t)=n_{\mathrm {eq}}(V_{j+1},T)+\delta n(t_{j+1})\exp (-\Lambda [t-t_{j+1}])). \end{aligned}$$

where $\delta n(t_{j+1})=\delta n(t_j)+n_{\mathrm {eq}}(V_j,T)-n_{\mathrm {eq}}(V_{j+1},T)$. The current can be obtained using Eq. (1) and calculated for any time *t*. At the beginning of the simulation we have to assign an initial population $n=n(t=0,V,T)$. The natural choice is $n(t=0,V,T)=n_{\mathrm {eq}}(V=0,T)$, which is the state of absolute equilibrium that would be achieved by waiting for an infinite time at zero voltage and finite temperature. But the initial condition has no effect on the hysteresis loop shown, which is established after the first cycle for any initial population.

In the simulated *I*–*V* curves shown at the bottom of Fig. 4, the voltage was swept stepwise from $-1.0$ to 1.0 V and back). We also took $T=300$ K, $\epsilon _1=2.7$eV, $\eta =1.2$, $\Delta U=0$, $q=0.5\mathrm {e}$, $\omega _1=10^{12}$ Hz. With these parameters, there is a complete population transfer at each end of the hysteresis loop, from $n=0$ to $n=1$ at positive *V* and from $n=1$ to $n=0$ at negative *V*. So, if we were to continue increasing the voltage beyond positive 1.0*V* (beyond negative $-1.0$ V), the curve would continue along the slope of the HRS (of the LRS).

It should be evident that this model has far too many parameters, most of which we only know to within a factor of unity, to take a quantitative fit between model prediction and experiment seriously. But it may be useful in providing an intuitive picture of the RS. For this purpose, it is helpful to consider the symmetric case $\eta =1$, $\Delta U=0$, ignore terms quadratic in $qV/\epsilon _1$, and take $kT=0$. The barrier is then at $X_b=\ell /2$ with height $U(X_b)=\epsilon _1/2+qV/2$ and valley energies are $U(X_1)=0$ and $U(X_2)=qV$. When $qV>0$, state 1 has a lower energy than state 2 but a bias $qV>\epsilon _1$ is needed before the system can overcome the barrier and switch from state 2 to 1. Similarly, a reverse bias $qV<-\epsilon _1$ is needed for transitions from 1 to 2. RS occurs because the system is trapped in alternating energy valleys as the voltage sweeps through $V=0$.

To test non-volatile memory for such a model, we interrupted the procession along the hysteresis loop at a given time, $t=t_i$ and voltage $V_s=V(t_i)$ when the current is $I(t_i)=G_2V_s$ by dropping *V* to 0. Then we continued to update the time-dependent current, using Eq. (1), for time $t_s$ before restarting *V*(*t*). But the transition rates $\Lambda _{1\rightarrow 2}$ and $\Lambda _{2\rightarrow 1}$ are exponentially small at zero voltage, being determined by a factor of order $\exp (-\epsilon _1/kT)$. So, the current is unaffected over any reasonable $t_s$ and memories effectively last forever.

**Figure 9:**
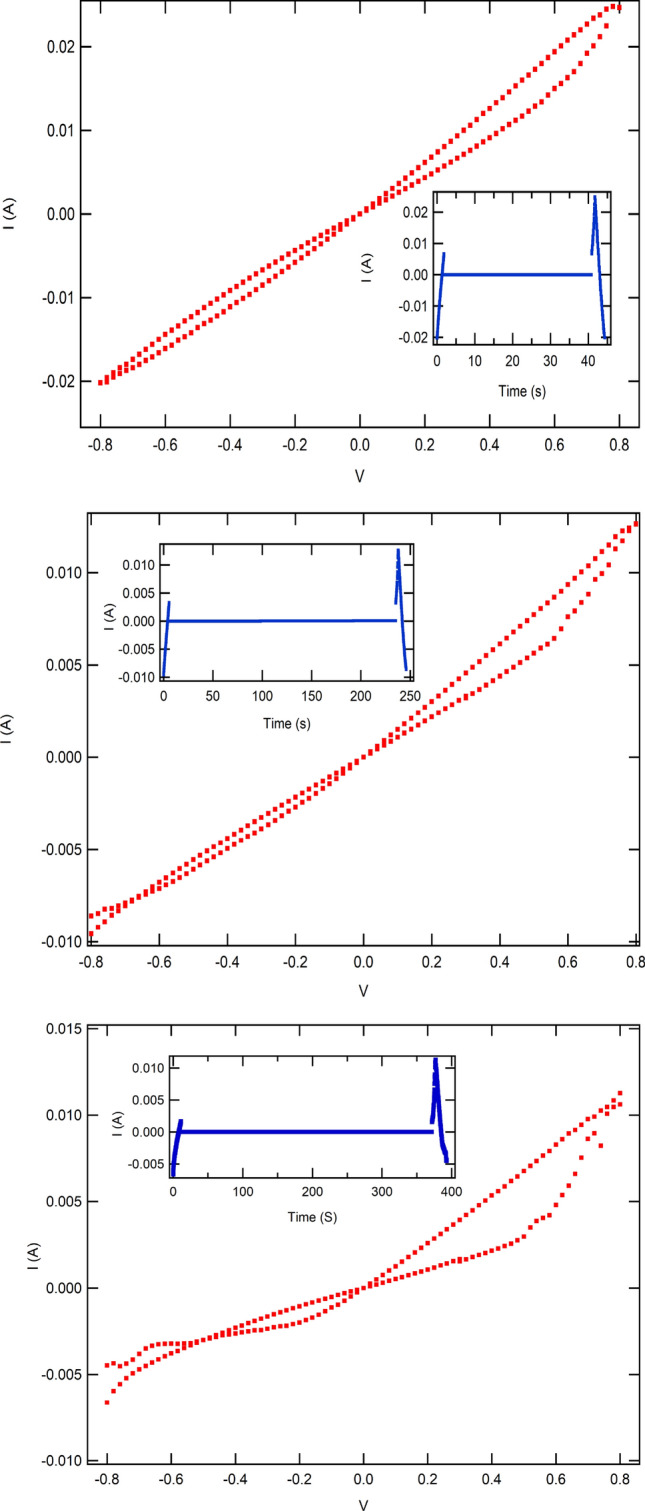
The main panels show *I*–*V* curves obtained after interrupting the sawtooth variation of voltage, dropping *V* to zero, and then waiting for time $t_s=40,240,360$ s from top to bottom. The insets show the time-dependence of the applied voltage *V*. For the first two panels, the hysteresis curve is essentially unchanged from that observed before the interruption. However, for $t_s=360$ s, the distortion of the *I*–*V* curve is evident.

The $t_s$-dependence of Fig. 3, which would limit memory retention, likely arises from effects far beyond this model. For example, alternative relaxation mechanisms between state 1 and 2 may dominate the direct transition given by Eq. (6) at $V=0$, whereas the latter is dominant at finite *V* and thus responsible for the hysteresis loop. In the simplistic picture offered by Eq. (2), the effective conductance of the diamondoid film must always be intermediate to the constant values of the pure LRS and HRS. The results of Fig. 9, on the other hand, indicate that the conductances of both states may be decreasing with time. We have not done an extensive study^46^ of memory-retention properties or examined in detail the time-dependent degradation of the RS. The main conclusion we draw from experiment is that two-state conduction for this system, with conductances $G_1$ and $G_2$ in a robust hysteresis loop, persists for several minutes but afterwards becomes unreliable.

## Conclusion

The electrical transport properties of diamondoid thin films, deposited on an Au/W substrate, were investigated experimentally. The *I*–*V* curves exhibit a hysteresis loop, with high-resistance and low-resistance states, characteristic of resistive switching. The observation of resistive switching in a putative monolayer, which has appealing material properties, is promising for applications to non-volatile computer memory. Though the microscopic mechanism for the observed RS is unknown, we provided a phenomenological model that captures the behavior of the measured hysteresis loop.

## Figures and Tables

**Figure 7:**
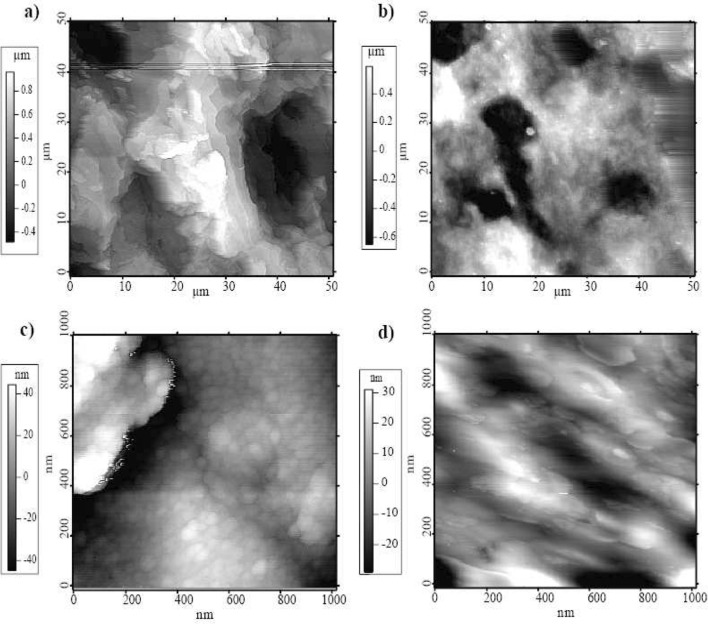
Atomic-force microscopy (AFM) images of the W/Au substrate without the diamondoid monolayer (**a**,**c**) and with the monolayer present (**b**,**d**). (**a**) and (**b**) show the top-view of a square region of length 50 $\upmu$m, while (**c**) and (**d**) show the top view of a square region with length 1 $\upmu$m.

## Appendix

Here we present additional data related to sample characterization and the retention-time of the *I*–*V* hysteresis loops. Note that the procedure used to fabricate the diamondoid monolayers, described above, is well-established and the resulting samples have been rigorously characterized (see^38^, references therein and citations thereof.) The diamondoid layer forms on the gold surface as a film that is one molecule thick and arranged in a hexagonal crystal structure. Below we partially characterize the samples using atomic force microscopy (AFM) and scanning electron microscopy (SEM) to confirm that the films have the expected gross features.

In Fig. 5, the current *I* measured between the Au/W substrate, with and without the diamondoid film on its surface, and the cat’s whisker lead is shown as a function of applied voltage *V*. For the bare substrate, $I=G_sV$ with $G_s\approx 0.4\,\,\Omega ^{-1}$. There is no sign of hysteresis. When the diamondoid film is present, the hysteresis loop is observed. This clearly indicates that it is the diamondoid film that is the source of the RS.

An example of an *I*–*V* data set is given in Fig. 6. The measurement begins with $V=0$, then the voltage is cycled up and down five times, as described above. The initial increase of *I* with *V* does not lie on the hysteresis curve, which is established after the first half cycle. This initial behavior, sensitive to the state of the system prior to measurement, is omitted when we construct hysteresis curves like those shown above. Note that the hysteresis loop, once established, is qualitatively the same each cycle. It should be evident that the HRS exhibits non-linear *I*(*V*) at small *V* in these curves. This suggests an obvious extension to the model given in the previous section: the HRS could be associated with a non-Ohmic conducting channel. However, for typical averaged *I*(*V*) curves, the simplified picture given above is sufficient.

Figure 7 presents atomic force microscopy (AFM) images of the top view of the substrate, with and without the diamondoid film present. The surface roughness for the diamondoid-coated samples, from Fig. 7d is a few nanometers. The adamantane molecule has a length of order 1 nm.

Figure 8 presents scanning electron microscopy (SEM) images of the top view of the substrate with the diamondoid present. The dark regions, indicating flakes of the diamondoid layer adsorbed on the gold surface, have a typical length scale of a few micrometers along the surface.

The AFM and SEM data shown here do not establish that the thin diamondoid film is a monolayer. They merely give some indication of the deposition pattern on the substrate surface.

Figure 9 provides a more detailed picture of the persistence of the NVM associated with the observed RS. As discussed previously, a hysteresis loop is observed when the voltage *V* is ramped up and down with time in a sawtooth pattern. To simulate powering down the device, we interrupted this time variation, dropped the voltage abruptly to zero, then waited for a time $t_s$. After this, we restored *V* to its previous value and resumed the sawtooth variation.

In Fig. 3, we showed the difference in the current value, measured just before and just after the interruption, as a function of $t_s$. The current change began to increase significantly for $t_s>200$ s or so. In the three panels of 9, we show the hysteresis loops observed after interruptions of various time $t_s$. Here it can be seen that the qualitative shape of the *I*–*V* loop obtained after an interruption of $t_s=40, 240$ s appears unchanged from that observed before interruption (i.e. from those loops shown above). When $t_s=360$ s the hysteresis loop, though remaining well-defined, is evidently distorted from its previous shape. These results again point to a retention time scale of order $t_s\gtrapprox 200$ s.
